# High-Barrier Polyimide Containing Carbazole Moiety: Synthesis, Gas Barrier Properties, and Molecular Simulations

**DOI:** 10.3390/polym12092048

**Published:** 2020-09-08

**Authors:** Yiwu Liu, Ao Tang, Jinghua Tan, Xianqing Zhao, Chengliang Chen, Ding Wu, Yuhui Li, Pan He, Hailiang Zhang

**Affiliations:** 1 National and Local Joint Engineering Center of Advanced Packaging Materials R & D Technology, Key Laboratory of Advanced Packaging Materials and Technology of Hunan Province, School of Packaging and Materials Engineering, Hunan University of Technology, Zhuzhou 412007, China; liuyiwu@hut.edu.cn (Y.L.); tangao1234@163.com (A.T.); zxq204565296@163.com (X.Z.); chenchengliangc@163.com (C.C.); wu923933134@163.com (D.W.); lynn18207491136@163.com (Y.L.); hp1846898110@163.com (P.H.); 2 Key Laboratory of Polymeric Materials and Application Technology of Hunan Province, Key Laboratory of Advanced Functional Polymer Materials of Colleges, Universities of Hunan Province, College of Chemistry, Xiangtan University, Xiangtan 411105, China; hailiangzhang@xtu.edu.cn

**Keywords:** polyimide, carbazole, barrier properties, molecular simulations

## Abstract

A high-barrier polyimide (2,7-CPI) was synthesized through the polymerization of pyromellitic dianhydride (PMDA) and a novel diamine (2,7-CDA) containing carbazole moiety. The synthesized diamine and polyimide were fully characterized by elemental analyses, FTIR and NMR. The 2,7-CPI displays very attractive barrier performances, with oxygen transmission rate (OTR) and water vapor transmission rate (WVTR) low to 0.14 cm^3^·m^−2^·day^−1^ and 0.05 g·m^−2^·day^−1^, respectively. Meanwhile, 2,7-CPI also exhibits exceptional thermal stability with a glass transition temperature (*T_g_*) of 467 °C, 5% weight-loss temperature (*T_d5%_*) of 550 °C under N_2_ and coefficient of thermal expansion (*CTE*) of 3.4 ppm/K. The barrier performances of 2,7-CPI are compared with those of a structural analogue (2,7-CPPI) and a typical polyimide (Kapton). Their barrier performances with respect to microstructure were investigated by molecular simulations, wide angle X-ray diffraction (WAXD), and positron annihilation lifetime spectroscopy (PALS). The results show that 2,7-CPI possesses better coplanar structure and more number of intermolecular hydrogen bonds among the three PIs, which result in tight chain packing and thereby high crystallinity, low free volume, and decreased chains mobility. That is, the high crystallinity and low free volume of 2,7-CPI reduce the diffusion and solubility of gases. Meanwhile, the poor chains mobility further decreases the gases diffusion. The reduced diffusion and solubility of gases consequently promote the improvement of barrier properties for 2,7-CPI. The polyimide has a wide application prospect in the field of flexible electronic packaging industries.

## 1. Introduction

In recent years, flexible displays have attracted considerable attention because of the low weight, high mobility, and their portable, wearable, and even foldable applications [1,2]. Flexible active matrix organic light emitting diode (AMOLED) display is widely accepted as the most promising development trend for flexible displays technology, because they show low power consumption, faster response, and are more power efficient [3,4]. Nowadays, plastic films are widely used as substrates for flexible AMOLED, owing to their light weight, good flexibility, and excellent robustness [5,6]. With the wide use of top-emitting architectures in flexible AMOLED, transparency is not a prerequisite for the substrates [7]. However, to realize the application of plastic substrates in AMOLED, two key requirements should first be satisfied for the plastic films. One is the high gas barrier properties (WVTR < 10^−6^ g·m^−2^·day^−1^ and OTR < 10^−5^ cm^3^·m^−2^·day^−1^), which can prevent the degradation of organic materials in the devices caused by the penetration of moisture and oxygen through the substrates [8,9]. The other is the high thermal and dimensional stability. As the driven device, high-performance thin-film transistors (TFTs) are crucial for AMOLED displays. High processing temperature is significantly important for the performance improvement of TFTs [10]. The widely used low-temperature-poly-silicon (LTPS) TFTs require the plastic substrates to withstand process temperature of higher than 400 °C [11]. In addition, high dimensional stability can prevent the substrates to shrink during the temperature cycles associated with the TFT fabrication, and guarantee good overlay alignment in the TFT array [12]. Hence, the plastic substrates are required to possess excellent thermal and dimensional stability (*T_g_* ≥ 400 °C, *CTE* ≤ 8 ppm/K) [9,11]. However, the demands for thermal and dimensional stability far exceed the tolerance range of conventional plastic substrates such as polyethylene terephthalate (PET) and polyethylene naphthalate (PEN) (*T_g_* ˂ 200 °C, *CTE* of 20~80 ppm/K) [13].

In this regard, polyimides (PIs) have been considered as one of the best choices for flexible AMOLED substrates because of their excellent thermal stability and mechanical properties [14,15]. However, the insufficient barrier performances of PIs affect their application in AMOLED substrates. To improve the barrier performances of PIs, inorganic barrier layers such as aluminum oxide and silicon dioxide or inorganic/organic alternating layers are usually fabricated on PIs by sputtering or chemical vapor deposition (CVD) [16,17]. However, the deposition methods are usually complicated, and require large-scale equipment, thus lead to fairly high fabrication costs. Apparently, these deposition processes are on the basis of intrinsic PI matrix. Hence, enhancing the barrier performances of intrinsic PIs can reduce the thickness of surface barrier layers [18], simplify the deposition processes and decrease costs.

**Figure 1:**
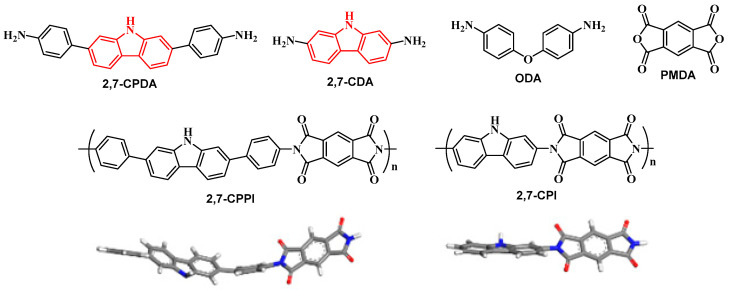
The chemical structure of 2,7-CPDA, 2,7-CDA, ODA, and PMDA and the stereo view of the lowest energy conformation of repeat units for 2,7-CPPI and 2,7-CPI.

Many researches reported that the gas permeability of polymers was greatly determined by their chemical structures, aggregation structures, and interchain cohesion [19,20]. Polyimides possess the advantage of high designability of molecular structures. In this case, structural modifications are considered as effective routes to enhance the barrier performances of PIs. In our previous work, a diamine (2,7-CPDA, see Figure 1) containing rigid planar carbazole moiety was synthesized and polymerized with PMDA to obtain polyimide (2,7-CPPI) [21]. The introduction of carbazole moieties increases the regularity and rigidity of polymer chains and the interchain cohesion, which improves the chains packing efficiency and thereby the barrier performances of 2,7-CPPI.

In this work, a novel diamine (2,7-CDA) containing rigid planar carbazole moiety was designed and synthesized. Then, the 2,7-CDA was polymerized with PMDA to form polyimide (2,7-CPI). The chemical structure of 2,7-CDA is shown in Figure 1. Compared with 2,7-CPDA, the two amine groups in 2,7-CDA are directly attached to the 2,7-positions of the carbazole ring without the bridging of benzene rings. The stereo view of the lowest energy conformations of repeat units for 2,7-CPI and 2,7-CPPI are presented in Figure 1. It can be observed that the diamine residues in 2,7-CPI exhibit a better coplanarity than those in 2,7-CPPI, which is conducive to the close chains packing. In addition, the direct connection of two large rigid planar structures (carbazole ring and dianhydride residue) in 2,7-CPI will increase the chains rigidity and reduce the mobility of polymer chains. These above-mentioned structural characteristics of 2,7-CPI are beneficial to the improvement of barrier performances.

Here, the synthesis, characterizations, as well as barrier and thermal properties of 2,7-CPI were reported. Additionally, the barrier performances of 2,7-CPI are compared with those of a typical polyimide (Kapton) based on diamine 4,4′-oxydianiline (ODA) and PMDA and our previous reported structural analogue 2,7-CPPI. Molecular simulation is an increasingly vital tool to provide detailed insights into the structural characteristics and gas transport performances of polymers at a microscopic level, which is hard to obtain by experiments [22,23]. In order to elucidate the effect of different diamine structures on the barrier properties of PIs, molecular simulations, WAXD and PALS were utilized to study the chains packing, intermolecular hydrogen bonds, free volume, chains mobility, penetrant trajectories, diffusivities, and sorption behavior of PIs. This will be helpful in revealing the barrier mechanism and developing high-barrier polymer membranes.

## 2. Experimental Section

### 2.1. Materials

O-aminobiphenyl, decalin, palladium 10% on activated carbon (10% Pd/C) were purchased from Energy-chemical Company (Shanghai, China). Hydrazine hydrate and sodium nitrite (NaNO_2_) were obtained from Aladdin Company (Shanghai, China). Sodium azide and concentrated sulfuric acid were purchased from Wuhan changcheng Chemical Technology Co., Ltd. (Wuhan, China). Potassium nitrate (KNO_3_), sodium hydroxide (NaOH), sodium chloride (NaCl), absolute ethanol (EtOH), N-hexane, methylene chloride, ethyl acetate, N,N-dimethylformamide (DMF), and silica gel were obtained from Sinopharm Group Chemical Reagent Co., Ltd. (Shanghai, China). ODA and PMDA were purchased from Alfa-Aesar company (Shanghai, China). Before use, the PMDA was dried at 110 °C under vacuum for 6 h and DMF was purified by distillation under inert nitrogen atmosphere.

### 2.2. Instrumentation 

All nuclear magnetic resonance spectra (NMR) were recorded on a Bruker ARX400 MHz spectrometer (Bruker Corporation, Fallanden, Switzerland). Samples were prepared as solution of 5~10 mg of compound in 0.5 mL of deuterated dimethyl sulfoxide (DMSO) using tetramethylsilane (TMS) as the internal reference. Mass spectra were measured on a AcquityUPLC/UPC2/Xevo G2-XS QTOFMS (Waters Corporation, Milford, MA, USA). Elemental analysis was carried out on a Vario EL cube Elemental Analyzer (Elementar Corporation, Langenselbold, Germany). Infrared spectra were recorded on a Nicolet iS10 Fourier-transform infrared (FT-IR) spectrometer (Thermo Fisher Scientific, Waltham, MA, USA). The molecular mass of the poly(amic acid) (PAA) was estimated by gel permeation chromatography on multi-angle laser light scattering (GPC-MALLS) system (Wyatt Technology Corporation, Santa Barbara, CA, USA). DMF is the eluent with flow rate of 1 mL/min and test temperature of 50 °C. Wide angle X-ray diffractograms (WAXD) were recorded by a Rigaku, Ultema III X-ray diffractometer (Rigaku Corporation, Tokyo, Japan) using a Cu Kα radiation. Density was obtained with an ALFA MIRAGE SD-200L electronic density balance (Alfa Mirage Corporation, Osaka, Japan).

Thermogravimetric analyses (TGA) were performed with a TA thermal analyzer (TGA55, TA instruments, New Castle, DE, USA) under N_2_ with a heating rate of 20 °C/min from 50 to 800 °C, and heated under flowing nitrogen (40 mL/min). The dynamic mechanical (DMA) spectra of the samples were obtained by using TA thermal analyzer (DMA Q850, TA instruments, New Castle, DE, USA). The specimens were analyzed in tensile mode at a constant frequency of 1 Hz, amplitude of 20 μm, and a temperature range from 25 to 500 °C at a heating rate of 5 °C/min. Thermal mechanical analysis (TMA) was used to study the coefficient of thermal expansion (CTE) of the film with a heating rate of 5 °C/min from 25 °C to 350 °C by TMA Q400 instrument (TA instruments, New Castle, DE, USA) under nitrogen.

Tensile test was performed on samples cut from 35~50 μm thick sheet and tested using SANS CMT6103 instrument (New Sansi Material Testing Co., Ltd. Shenzhen, China) according to GB/T16421-1996. The specimen size is 10 × 100 mm, Jaw separation is 50 mm. Jaw speed was first set to 2 mm/min, when elongation reached 1 mm, the Jaw speed was changed to 20 mm/min.

The oxygen permeability of a PI film was measured by Mocon (Mocon Corporation, Minneapolis, MN, USA), in accordance with ASTM-D 3985, using an OX-TRAN 2/21 ML instrument at 23 °C and 0% RH. The moisture vapor permeability was measured using the model PERMATRAN-W^®^ 3/33 of the Mocon Corporation (Indio, CA, USA) at 90% RH and 37.8 °C according to ASTM F-1249. The specimen of 75 μm was fixed on an aluminum foil with the film testing area of 5 cm^2^.

Positron lifetime measurements were performed as follows: two identical samples with dimension of 1.5 × 10 × 10 mm^3^ were sandwiched with a 10 μCi ^22^Na positron source. The ^22^Na nucleus emits a 1.28 MeV γ-ray simultaneously (within a few ps) with the positron. The positron lifetime is determined from the time delay between the emission of the birth gamma (1.28 MeV) and one of the 0.511 MeV annihilation photons. Lifetime measurements were carried out using a fast-fast coincidence system with a time resolution of about 210 ps and a channel width of 12.6 ps. We have analyzed the lifetime spectra with the help of the data processing programs PATFIT. Before analyzing each spectrum, the positron source components (377 ps/11.75%, 1.04 ns/0.17%) were subtracted. The variance of the fits was around 1.1.

### 2.3. Synthesis of 4,4′-Dinitro-[1,1′-Biphenyl]-2-Amine (DPNA)

O-aminobiphenyl (4.231 g, 25 mmol) and concentrated sulfuric acid (50 mL) were added to a 100-mL three-necked flask under argon atmosphere. Then, KNO_3_ (5.303 g, 52.5 mmol) was added with stirring at 0 °C for 4 h. After that, the reaction mixture was poured into ice water. NaOH was added to adjust the solution to neutral. Subsequently, the orange intermediate (DPNA) was obtained through filtration, which was vacuum-dried at 80 °C for 24 h. The yield of the product was about 75 % (4.860 g). IR (KBr, *v*, cm^−1^): 3380 (-NH_2_ stretching), 1507 (−NO_2_ stretching), 1260 (C−N stretching), 1100~700 (Ar–H stretching). ^1^H NMR (400 MHz, DMSO-*d*_6_, *δ*, ppm): 8.31 (t, *J* = 12.8 Hz, 2H), 7.85 – 7.61 (m, 3H), 7.45 (d, *J* = 7.9 Hz, 1H), 7.28 (d, *J* = 8.1 Hz, 1H), 5.75 (s, 2H). ^13^C NMR (100 MHz, DMSO-*d*_6_, δ, ppm): 148.79, 147.29, 147.22, 145.18, 131.76, 130.48, 129.60, 124.49, 110.95, 109.66. MS (EI, *m*/*z*): 259(100) ([M^+^], calcd for C_12_H_9_N_3_O_4_, 259.06). Anal. Calcd for C_12_H_9_N_3_O_4_: C, 55.60; H, 3.50 and N, 16.21; found: C, 55.53; H, 3.52 and N, 16.24.

### 2.4. Synthesis of 2-Azido-4,4′-Dinitro-1,1′-Biphenyl (DPNN_3_)

Sulfuric acid (10 mL, 6 mol/L) was added into a three-necked flask, and then cooled to 0 °C to remove oxygen. After that, DPNA (0.518 g, 2 mmol) was added with stirring at 0 °C for 0.5 h. Subsequently, NaNO_2_ (0.207 g, 3 mmol) and NaN_3_ (0.195 g, 3 mmol) aqueous solutions with a concentration of 1 mol/L were slowly added with stirring at 0 °C for 3 h. Then, the intermediate (DPNN_3_) was extracted by 30 mL of dichloromethane, and washed by saturated salt water to remove impurities. The yield of the product was about 72% (0.411 g). IR (KBr, *v*, cm^−1^): 2127 (R-N=N=N stretching), 1509 (−NO_2_ stretching), 1347 (C−N stretching), 1100~700 (Ar–H stretching). ^1^H NMR (400 MHz, DMSO-*d*_6_, *δ*, ppm): 8.33 (d, *J* = 8.7 Hz, 2H), 8.16 (s, 1H), 8.14 – 8.06 (m, 1H), 7.83 (d, *J* = 8.7 Hz, 2H), 7.73 (d, *J* = 8.4 Hz, 1H). ^13^C NMR (100 MHz, DMSO-*d*_6_, *δ*, ppm): 148.51, 147.77, 142.87, 139.20, 136.83, 132.65, 131.29, 123.86, 120.25, 115.22. MS (EI, *m*/*z*): 285(10) ([M^+^], calcd for C_12_H_7_N_5_O_4_, 285.05). Anal. Calcd for C_12_H_7_N_5_O_4_: C, 50.53; H, 2.47 and N, 24.55; found: C, 50.49; H, 2.50 and N, 24.59.

### 2.5. Synthesis of 2,7-Dinitro-9H-Carbazole (2,7-CDN)

The mixture of DPNN_3_ (0.285 g, 1 mmol) and decalin (15 mL) was placed in a three-necked flask under argon atmosphere, and then heated to 155 °C with stirring for 6 h. After cooling down to room temperature, the product (2,7-CDN) was concentrated and purified by silica-gel column chromatography using ethyl acetate/n-hexane (*v*/*v* = 1/3), and then vacuum-dried at 80 °C for 24 h. The yield of the product was about 65 % (0.167 g). IR (KBr, *v*, cm^−1^): 1510 (−NO_2_ stretching), 1345 (C−N stretching), 3379 (N-H stretching), 1100~700 (Ar–H stretching). ^1^H NMR (400 MHz, DMSO-*d*_6_, *δ*, ppm): 12.23 (s, 1H), 8.47 (d, *J* = 8.8 Hz, 4H), 8.07 (dd, *J* = 8.7, 1.8 Hz, 2H). ^13^C NMR (100 MHz, DMSO-*d*_6_, *δ*, ppm): 147.08, 141.28, 126.47, 123.08, 115.02, 108.40. MS (EI, *m*/*z*): 257(100) ([M^+^], calcd for C_12_H_7_N_3_O_4_, 257.04). Anal. Calcd for C_12_H_7_N_3_O_4_: C, 56.04; H, 2.74 and N, 16.34; found: C, 56.00; H, 2.78 and N, 16.36.

### 2.6. Synthesis of 9H-Carbazole-2,7-Diamine (2,7-CDA)

2,7-CDN (2.572 g, 10 mmol) and absolute ethanol (450 mL) were added into a three-necked flask with stirring for 0.5 h at room temperature under argon atmosphere to remove oxygen. Then, the mixture was heated to 80 °C, and Pd/C (0.02 g) and hydrazine hydrate (5 mL) were added with stirring at 80 °C for 24 h under argon atmosphere. After cooling to room temperature, the Pd/C was removed by filtration and the grey white diamine (2,7-CDA) was obtained by lower temperature crystallization. Yield: 93 % (1.834 g). IR (KBr, *v*, cm^−1^): 3390 (N-H stretching), 3318 (-NH_2_ stretching), 1617 (*δ* N-H), 1273 (C-N stretching), 1100~700 (Ar–H stretching). ^1^H NMR (400 MHz, DMSO-*d*_6_, *δ*, ppm): 10.24 (s, 1H), 7.43 (d, *J* = 8.2 Hz, 2H), 6.49 (d, *J* = 1.7 Hz, 2H), 6.34 (dd, *J* = 8.2, 1.8 Hz, 2H), 4.87 (s, 4H). ^13^C NMR (100 MHz, DMSO-*d*_6_, δ, ppm): 146.09, 141.63, 119.10, 114.89, 107.69, 95.39. MS (EI, *m*/*z*): 197(100) ([M^+^], calcd for C_12_H_11_N_3_, 197.10). Anal. Calcd for C_12_H_11_N_3_: C, 73.07; H, 5.62 and N, 21.30; found: C, 73.02; H, 5.65 and N, 21.32.

### 2.7. Synthesis of Polyimides

In clean rooms, the polyimide (2,7-CPI) was synthesized following the procedure: 2,7-CDA (0.3379 g, 1.713 mmol) was dissolved in 10 mL of DMF in a 50-mL flask under argon atmosphere. Then, PMDA (0.3736 g, 1.713 mmol) was added, reaching a solid content of about 7 wt%. The reaction mixture was stirred at 0 °C for 8 h to produce a viscous poly(amic acid) (PAA) solution. Then, the PAA is defoamed and coated uniformly on a glass plate. Subsequently, the thermal imidization was carried out in a vacuum oven with the temperature program of 100 °C (1 h) / 100~200 °C (1 h) / 200~300 °C (1 h)/300~400 °C (1 h) to form 2,7-CPI film. The 2,7-CPI film was then released from the glass plate after the oven cooled down to room temperature. IR (KBr, *v*, cm^−1^): 1775 and 1713 (C=O stretching), 1359 (C–N stretching), 1100~700 (Ar–H stretching). Anal. Calcd for C_22_H_9_N_3_O_4_: C, 69.66; H, 2.39 and N, 11.08; found: C, 69.25; H, 2.55 and N, 11.41.

### 2.8. Molecular Simulation 

#### 2.8.1. Construction of Polymer Microstructures 

Biovia Materials Studio software was employed to perform simulations in this study using the COMPASS (Condensed-phase Optimized Molecular Potentials for Atomistic Simulation Studies) forcefield [24,25]. Initially, polymer chains containing 25 repeat units were built and geometry optimization was performed. Then, a periodic model of the PI, comprising of five polymer chains in cubic unit cell, was constructed and the total energy of the system was minimized using smart minimizing method. Annealing was performed by the NPT (constant number of particles (N), pressure (P), and temperature (T)) dynamics procedure through heating and cooling the system at 1 atm in the temperature range of 300 to 1000 K in steps of 50 K. Interactions of non-bond, van der Waals, and electrostatic forces, were calculated using an atom-based summation method and an Ewald summation method, respectively. The annealed cell was later put through a stage-wise equilibration procedure. First the cell was heated to 1000 K, and then the temperature was decreased in several stages to 600 K in steps of 100 K. After this, the temperature was decreased in several stages to 400 K in steps of 50 K and then decreased to 300 K in steps of 25 K. Each stage consisted of two consecutive NVT (constant number of particles (N), volume (V) and temperature (T)) and NPT runs at 1 atm and a specific temperature. The aim of the procedure was to obtain a refined system that would relax at the experimental density of the amorphous polymer at 1 atm and 300 K. Finally, the cell was relaxed by consecutive NVT (at 300 K) and NPT dynamics (at 1 atm and 298 K) to ensure that a constant density has been reached. Two criteria were used to determine the equilibrium of the system: (1) The density of the system remained stable for a long time; (2) the fluctuation of energy was lower than 10 % [26]. The plots of density and energy versus simulation time in the last NPT for PI systems are shown in supporting information Figures S1 and S2. It can be seen that the PI systems have reached equilibrium states. The equilibrated densities of Kapton, 2,7-CPPI and 2,7-CPI systems are 1.41, 1.56, and 1.57 g/cm^3^, respectively, which are fairly close to the experimental densities (1.42, 1.57 and 1.58 g/cm^3^). In all runs, Nosé method was used for temperature control [27]. In NPT runs, the pressure was controlled by Berendsen’s method. During these simulations, the cutoff for the nonbonded interactions was taken as 15.5 Å.

#### 2.8.2. Free Volume

The free volume was determined by a grid scanning method using the Connolly task. The void distribution was estimated by a method previously used for micro-crystalline materials [28,29]. Specifically, the simulation cell was divided into three-dimensional fine grids with a size of approximately 0.25 Å. The void size at a grid was determined as the diameter of the maximum cavity that encloses the grid and additionally has no overlap with any polymer atom.

#### 2.8.3. Radial Distribution Functions

The radial distribution functions (RDF) refer to a measure of the probability that, given the presence of an atom at the origin of an arbitrary reference frame, there will be an atom with its center located in a spherical shell of infinitesimal thickness at a distance r from the reference atom. The RDF was calculated by the average of the static relationship of every given pair of particles AB using the following equation [30]:

(1) $$g_{AB}\left(r\right)=\frac{\langle n_{AB}\left(r\right)\rangle }{4\pi r^{2}\Delta \rho_{AB}}$$

where $\langle n_{AB}\left(r\right)\rangle$ is the average number of atom pairs between r and r+Δr, and $\Delta \rho_{AB}$ is the density of atom pairs of type AB.

#### 2.8.4. Diffusion Coefficients

The diffusivity of gas molecules through PI was estimated by first inserting ten molecules of each gas into the equilibrated simulation box. Then, minimization of the potential energy was performed using “smart minimizing method” run. After this, the cell was put through annealing and stage-wise equilibration procedures using the same parameters as described before. The resulting structure was then equilibrated by NVT and NPT simulations at 298 K in order to ensure that its minimized total energy remained approximately constant with respect to the simulation time. An NVE simulation of the system was performed for 10,000 ps. The diffusion coefficients can be calculated by means of the Einstein relation [31,32]:

(2) $$D=\frac{1}{6N}\underset{t\to \infty }{\lim}\frac{d}{dt}\langle \sum_{i}^{N}{\left|r_{i}\left(t\right)-r_{i}\left(0\right)\right|}^{2}\rangle$$

where *N* is the number of penetrants, $r_{i}\left(0\right)$ and $r_{i}\left(t\right)$ are the initial and final positions of the center of mass of penetrant *i* over the time interval t, and $\langle {\left|r_{i}\left(t\right)-r_{i}\left(0\right)\right|}^{2}\rangle$ is the averaged mean-square displacement (MSD) of the penetrant. The diffusion coefficient was determined from the slope of MSD versus time data. In this work, MSD of H_2_O and O_2_ were calculated from the trajectories of ten penetrant molecules in the PI microstructures.

#### 2.8.5. Determination of Local Mobility of Polymer Chains 

The backbone mean square displacements (MSD) of PIs were determined to investigate the local mobility of PI chains. An NVE simulation of the equilibrated system was performed for 5000 ps to study the backbone MSD.

#### 2.8.6. Dihedral Angle Profiles

Dihedral angle profiles are used to examine the mobility of linking groups of polymer chains. Four consecutive atoms in the backbone are used to calculate the dihedral angle *θ*. The dihedral angle is defined as the angle between the two planes formed by the first three and last three atoms. For the most extended planar conformation, the dihedral angle *θ* is ±180°. Figure S3 illustrates the schematic diagrams of the analyzed atomic segments for the dihedral angle analysis.

#### 2.8.7. Sorption Isotherm 

The equilibrated cell was used for grand canonical Monte Carlo (GCMC) simulations employing the standard Metropolis algorithm using the “Sorption Isotherm” module [33]. Both the polymer framework and the penetrant molecules were treated as rigid bodies. The degrees of freedom of the system were accordingly specified by the center-of-mass position and orientation of the molecules. Metropolis sampling was used for inserting or deleting permeant molecules as well as accepting or rejecting their translational and rotational configurational moves. The COMPASS force field and force field assigned partial charges on atoms were used. A VTμ simulation was performed at each fixed pressure and 298 K. The pressure of the penetrant gas was varied from 10 to 3000 kPa. For each pressure value, 10^5^ equilibration steps were first performed to ensure proper relaxation of the polymer chains in response to the insertion of the penetrant molecule, following which 10^6^ steps of production run were carried out. The sorption isotherm can be obtained in the form of a plot of the concentration of sorbed gas, C, as a function of pressure at constant temperature. The solubility coefficient, S, is then obtained from the limiting slope of the sorption isotherm at zero pressure as [34]:

(3) $$S=\underset{p\to 0}{\lim}\left(\frac{C}{p}\right)$$

where C is in units of cm^3^(STP)/cm^3^(polymer) and p is pressure.

## 3. Results and Discussion 

### 3.1. Synthesis and Characterization of Monomers and Polyimides

**Figure 2:**
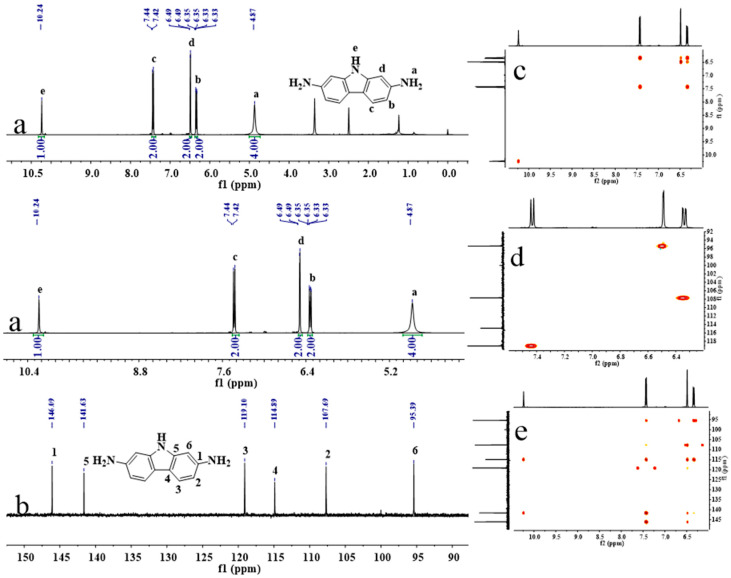
(**a**) ^1^H NMR (above: the spectra in the range of 0~11; below: the partial enlargement in the range of 4.5~11), (**b**) ^13^C NMR, (**c**) H-H COSY, (**d**) C-H QC and (**e**) C-H BC spectra of 2,7-CPA in DMSO-*d*_6_.

**Scheme 1:**
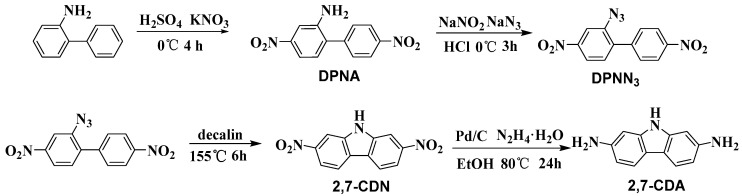
Synthesis route of 2,7-CDA.

Scheme 1 presents the synthesis route of 2,7-CDA. First, DPNA was prepared from o-aminobiphenyl by nitrification reaction. Second, DPNN_3_ was synthesized from DPNA by azide reaction. Subsequently, the as-obtained DPNN_3_ was reacted with decalin to form 2,7-CDN by cyclization reaction, which was then reduced to diamine 2,7-CDA. NMR, FT-IR, mass spectra, and elemental analysis were used to characterize the structures of the diamine and intermediate. The NMR, MS spectra of the intermediates DPNA, DPNN_3_, and 2,7-CDN and MS spectrum of 2,7-CDA are presented in supporting information Figures S4–S7. The NMR results of 2,7-CDA are given in Figure 2. The FT-IR spectra of diamine and intermediate are shown in Figure S8. The elemental analysis results are shown in experimental section. All these results were consistent with the proposed molecular structure of diamine and intermediate, confirming the successful synthesis of diamine by the mentioned four-step reactions.

**Scheme 2:**
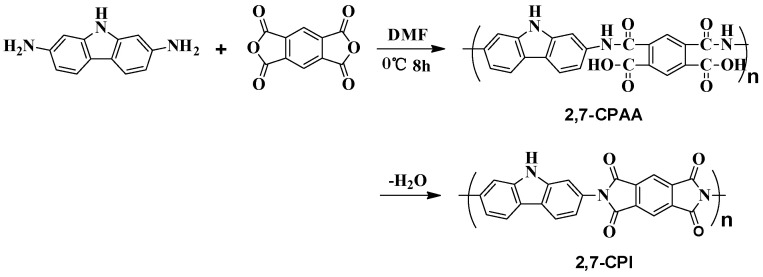
Synthesis route of polyimide.

The conventional two‒step thermal imidization method was used to prepare polyimide, as shown in Scheme 2. The 2,7-CDA was reacted with PMDA in DMF to form a precursor PAA, which was then converted to 2,7-CPI by thermal cyclodehydration. GPC was employed to measure the molecular weight of PAA. The weight-average molecular weight (*M*_w_) of the PAA was 3.67 × 10^5^, with a polydispersity index (*M_w_*/*M_n_*) of 1.96. The FT-IR was used to confirm the formation of 2,7-CPI in Figure S8. Compared with 2,7-CDA, the absorption peaks at 3318–3390 cm^−1^ (N‒H stretching) and 1617 cm^−1^ (*δ* N‒H) disappeared in 2,7-CPI, meanwhile the characteristic peaks of the imide group at 1359 cm^−1^ (stretching of C‒N) and 1775, 1713 cm^−1^ (stretching of carbonyl) were observed, suggesting the successful reaction between 2,7-CDA and PMDA, and the complete imidization reaction of the PAA.

### 3.2. Thermal and Mechanical Properties

**Figure 3:**
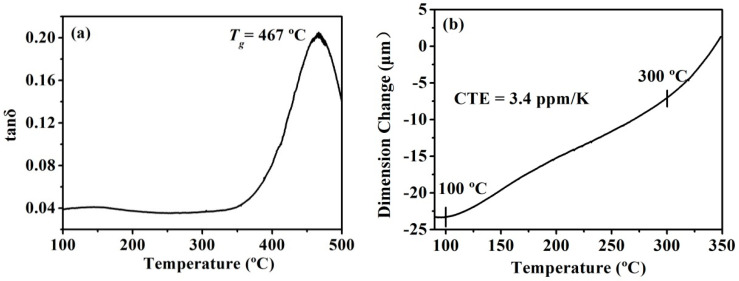
(**a**) DMA and (**b**) TMA curves of the 2,7-CPI film.

The thermal performances of 2,7-CPI were studied by DMA, TMA, and TGA, which are also compared with those of a typical polyimide (Kapton) and our previous reported structural analogue 2,7-CPPI. The results are listed in Table 1. The 2,7-CPI displayed the highest *T_g_* of 467 °C among the three PIs (Figure 3a). Additionally, the 2,7-CPI possessed outstanding dimensional stability, with a *CTE* low to 3.4 ppm/K in the range of 100~300 °C (Figure 3b). Furthermore, the 2,7-CPI exhibited exceptional thermal stability, with *T_d5%_* and *T_d10%_* of 550 °C and 590 °C, respectively (Figure S9). The tensile strength and modulus of 2,7-CPI were 134 MPa and 5.7 GPa, respectively. The dimensional and thermal stability and mechanical properties of 2,7-CPI were comparable to those of 2,7-CPPI, but better than Kapton. The excellent thermal and mechanical performances of 2,7-CPI and 2,7-CPPI were mainly attributed to the aromatic rigid carbazole moieties in the PI main chains and the strong interchain cohesion. Also, the 2,7-CPI had good flexibility, which was indicated from the rolled film in Figure S10. The excellent comprehensive performances of 2,7-CPI and 2,7-CPPI make them promising materials in the micro-electronics packaging industry.

### 3.3. Barrier Properties

The barrier performances of 2,7-CPI film are compared with Kapton and 2,7-CPPI, as shown in Table 2. Herein the diamine ODA used for the synthesis of Kapton has an ether bond between the two benzene rings (see Figure 1). The 2,7-CPI showed better barrier performances than Kapton and 2,7-CPPI. Its WVTR and OTR were low to 0.05 g·m^−2^·day^−1^ and 0.14 cm^3^·m^−2^·day^−1^, respectively, which reduced by three orders of magnitude in comparison with Kapton. Compared with 2,7-CPPI, the WVTR and OTR of 2,7-CPI showed reductions of 50% and 30%, respectively. The 2,7-CPI also showed superior barrier properties in comparison with most reported intrinsic polyimides [36,37] and other barrier polymers such as PET [38], ethylene-vinyl alcohol copolymer [39], nylon-6 [40], and polyvinylidene chloride [41].

### 3.4. Aggregation Structures Analysis 

**Figure 4:**
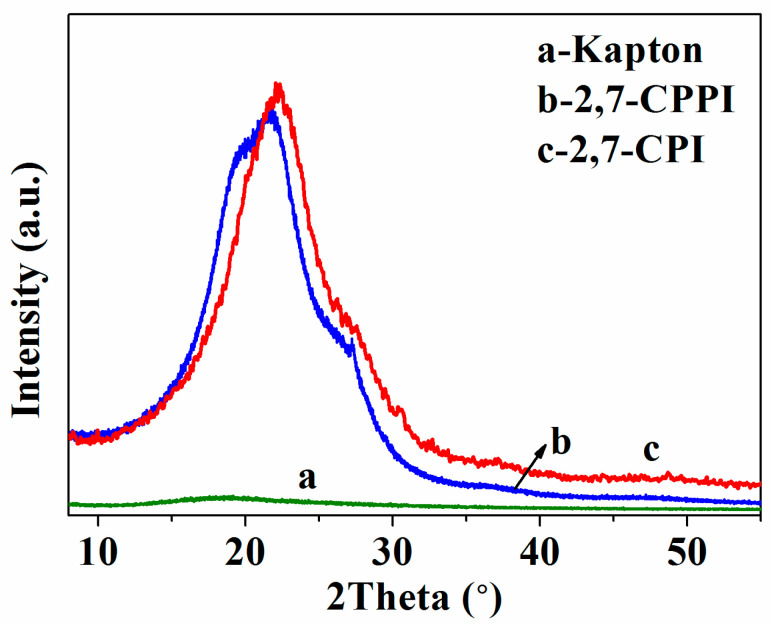
WAXD curve of Kapton, 2,7-CPPI and 2,7-CPI films.

In order to reveal the better barrier properties of 2,7-CPI in comparison with Kapton and 2,7-CPPI, WAXD was carried out to study the packing degree and order of polymer chains. The experimental WAXD scattering spectra of the three PIs are presented in Figure 4. The 2,7-CPPI and 2,7-CPI presented strong diffraction peaks, suggesting their higher crystallinity, while the broad bands of the Kapton demonstrated the amorphous nature of the polymer. The crystals in 2,7-CPPI and 2,7-CPI matrices will hinder the permeation of H_2_O and O_2_ by forcing them to follow a more tortuous diffusion pathway, and thereby improving their barrier properties [42]. The peak in WAXD patterns is ascribed to the chain-to-chain distance (*d*-spacing) between the polymer backbones [43]. The 2,7-CPI showed an intensive reflection peak at 22.11° corresponding to the *d*-spacing of 4.01 Å, which was lower than those of Kapton and 2,7-CPPI (Table 3). In addition, 2,7-CPI presented higher density than Kapton and 2,7-CPPI, as shown in Table 3. The low *d*-spacing and high crystallinity and density suggested the close chains packing of 2,7-CPI. The Kapton displayed lowest density and largest *d*-spacing among the three PIs, demonstrating its loose chains packing. This was mainly due to the nonplanar structure of ODA caused by the ether bond.

### 3.5. Hydrogen Bonds Analysis 

**Figure 5:**
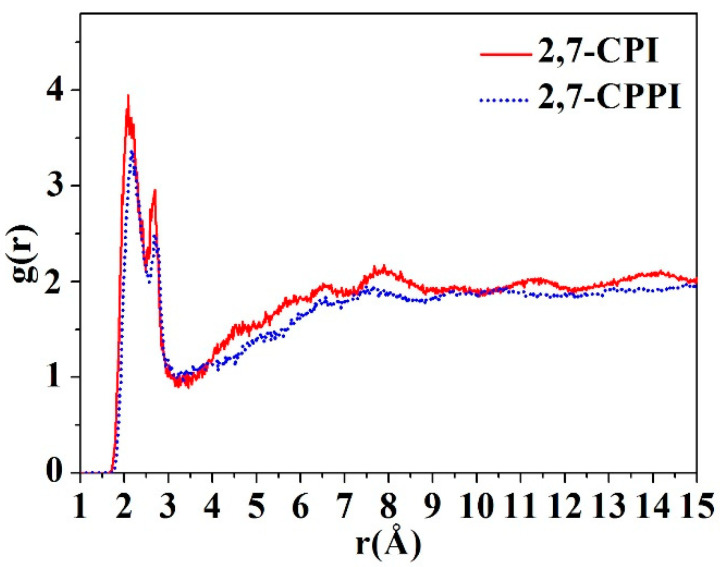
The RDFs of the hydrogen atoms of ‒HN‒ in carbazole and oxygen atoms of O=C‒ in imide rings for 2,7-CPPI and 2,7-CPI.

**Figure 6:**
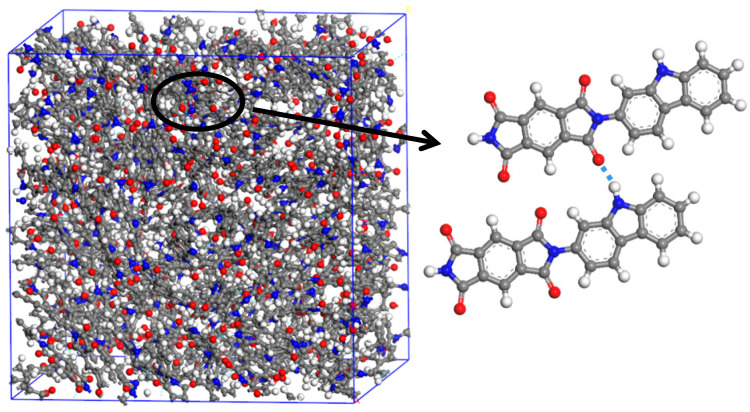
H-bonds between ‒HN‒ and O=C‒ in imide rings in simulation cell of 2,7-CPI. (red: oxygen atoms; blue: nitrogen atoms; white: hydrogen atoms; grey: carbon atoms).

Intermolecular interactions play an important role in chains packing. The radial distribution functions (RDFs) were calculated to study the intermolecular H-bonding interactions in PI matrices. In theory, the hydrogen bonds between ‒NH‒ of carbazole and O=C‒ of imide rings could be formed in the 2,7-CPPI and 2,7-CPI matrices, while no hydrogen bonds could be formed in Kapton. The radial distribution functions (*g_AB_(r)*) of 2,7-CPPI and 2,7-CPI were calculated to analyze the hydrogen atoms of ‒HN‒ in carbazole and the oxygen atoms of O=C‒ in imide rings, and the relevant calculated results are shown in Figure 5. Intermolecular interactions can be classified into H-bonding, strong and weak van der Waals’ forces, corresponding to the distances between atoms of 2.6~3.1, 3.1~5.0, and above 5.0 Å, respectively [44]. In Figure 5, there were peaks appearing in the range of 2.6~3.1 Å, suggesting the formation of hydrogen bonds in 2,7-CPPI and 2,7-CPI. Notably, 2,7-CPI exhibited higher *g(r)* value than 2,7-CPPI in that range, indicating the larger number of hydrogen bonds in 2,7-CPI compared to that in 2,7-CPPI. Figure 6 presents the hydrogen bonds formed in the simulation cell of 2,7-CPI, confirming the existence of the above-mentioned hydrogen bonds. Statistical results showed that 49 and 60 hydrogen bonds were formed in the 2,7-CPPI and 2,7-CPI systems, respectively (Table 3), while no hydrogen bonds were found in the Kapton system. The cohesive energy density (CED) of the three PIs systems were calculated by molecular simulations and shown in Table 3. The 2,7-CPI showed the highest CED among the three PIs, probably owing to the more number of hydrogen bonds formed in 2,7-CPI matrix. The strong intermolecular hydrogen bonding of 2,7-CPI contributed to its close chain packing. The low CED value of Kapton was also a reason for its loose chains packing.

### 3.6. Free Volume and Cavity Size Distribution Analysis

**Figure 7:**
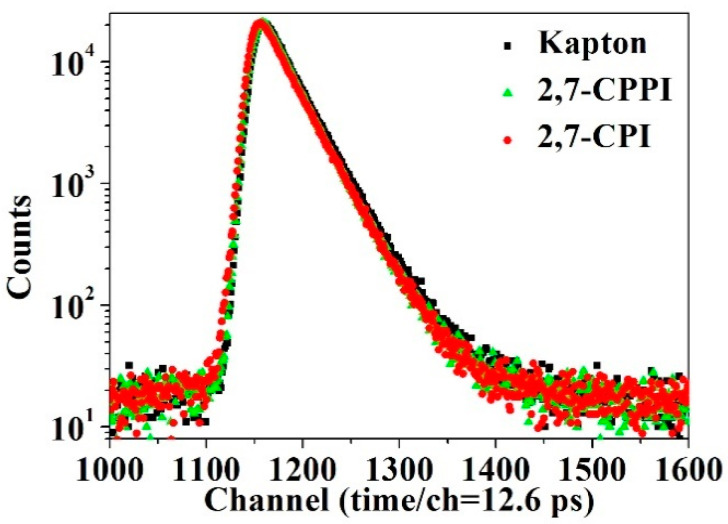
Positron lifetime curve measured for Kapton, 2,7-CPPI and 2,7-CPI films.

The above-discussed chain packing and interaction will affect the free volumes formed in PI matrix, which play a vital role in the gas transport performances. PALS was utilized to study the free volume of the PI films. The positron lifetime curves of Kapton, 2,7-CPPI and 2,7-CPI film are shown in Figure 7. The slope of the decay curve for Kapton was lower than those of 2,7-CPPI and 2,7-CPI, indicating the larger size of the free volume in Kapton [45]. The raw data obtained were resolved into two finite lifetime components using the PATFIT program which assumes a Gaussian distribution for the logarithm of the lifetime for each component. The obtained data of positron lifetime are listed in Table S1. Jean et al. have reported a relation between the second lifetime component *τ_2_* and the mean free-volume radius (*R*) of polymeric materials [46]. According to such correlation, the *R* of PI are calculated and shown in Table 3. In Table 3, the relative fractional free volume (*FFV*) is also calculated according to our previous work [21]. The *FFV* of the three PIs decreased in the order: Kapton ˃ 2,7-CPPI ˃ 2,7-CPI.

**Figure 8:**
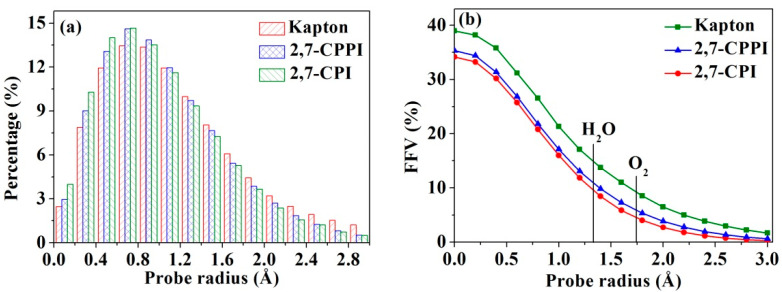
Distributions of void radius (**a**) and fractional free volume (**b**) as a function of probe radius in Kapton, 2,7-CPPI and 2,7-CPI. The kinetic radii of O_2_ and H_2_O are indicated by vertical lines.

Molecular simulations were used to study the characteristics of free volumes including size distribution, three-dimensional arrangement, and connectivity. The distributions of void size in Kapton, 2,7-CPPI and 2,7-CPI are shown in Figure 8a. Compared to Kapton, the 2,7-CPPI and 2,7-CPI had fewer voids with radius bigger than 1.2 Å. The kinetic radii of O_2_ and H_2_O are 1.73 Å and 1.325 Å, respectively, which are larger than 1.2 Å. This suggested that 2,7-CPPI and 2,7-CPI had fewer voids available for the diffusion of H_2_O and O_2_. In addition, 2,7-CPI possessed fewer voids with radius bigger than 0.8 Å in comparison with 2,7-CPPI, indicating the fewer number of large-size free volumes in 2,7-CPI.

**Figure 9:**
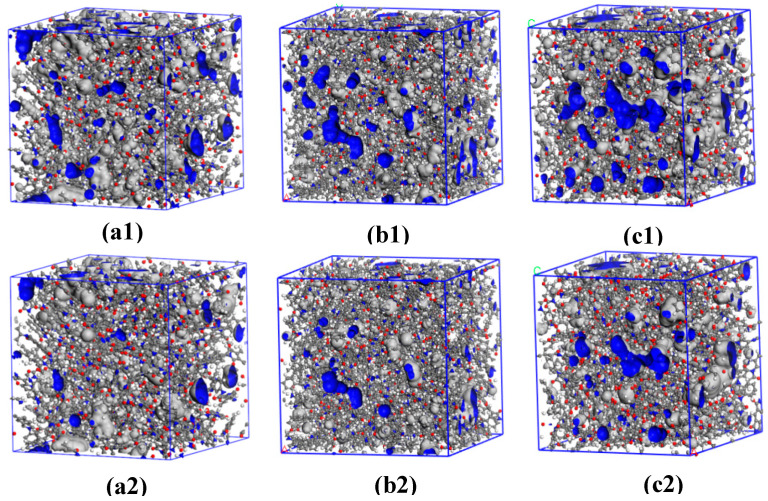
Three-dimensional representation of H_2_O and O_2_ accessible volume for (**a1**) (**a2**) 2,7-CPI, (**b1**) (**b2**) 2,7-CPPI and (**c1**) (**c2**) Kapton. (grey: Van der Waals surface; blue: Connolly surface; a1, b1 and c1: probe radius of 1.325 Å; a2, b2 and c2: probe radius of 1.73 Å).

In order to understand the available free volume for various penetrants, the *FFV* was analyzed using spherical probes with different radii. Figure 8b illustrates the *FFV* as a function of probe radius for Kapton, 2,7-CPPI and 2,7-CPI. The total fractional free volume (*FFV*^0^) for the three PIs was obtained when the probe radius was set as 0 Å. As expected, the *FFV* decreased with an increase in the probe radius. It is observed that 2,7-CPI showed the lowest *FFV* among the three PIs in all the related probe radius range. For reference, the kinetic radii of O_2_ and H_2_O considered in this study are indicated on the figure. The 2,7-CPI presented the lowest *FFV*^0^, H_2_O accessible *FFV* and O_2_ accessible *FFV*, as shown in Table 3. This was consistent with the *FFV* results of PALS. Furthermore, the morphological representation of the O_2_ and H_2_O accessible volumes in Kapton, 2,7-CPPI and 2,7-CPI are shown in Figure 9. The voids in 2,7-CPI were smaller and discontinuous compared with those of Kapton and 2,7-CPPI. This was due to the close chain packing caused by the better coplanar structures and strong intermolecular hydrogen bonding of 2,7-CPI. The less connected and low accessible free volume of 2,7-CPI resulted in low gas permeability and thereby high gas barrier. The Kapton showed larger and well-connected voids resulting from the nonplanar structure and low interchain cohesion, which led to high gas permeability.

### 3.7. Local Mobility of Polymer Chains

**Figure 10:**
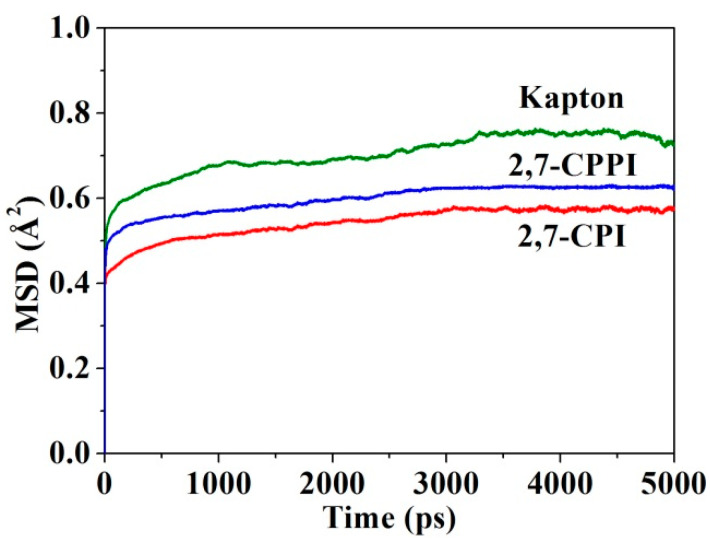
MSD of polymer chains as a function of time in Kapton, 2,7-CPPI and 2,7-CPI.

**Figure 11:**
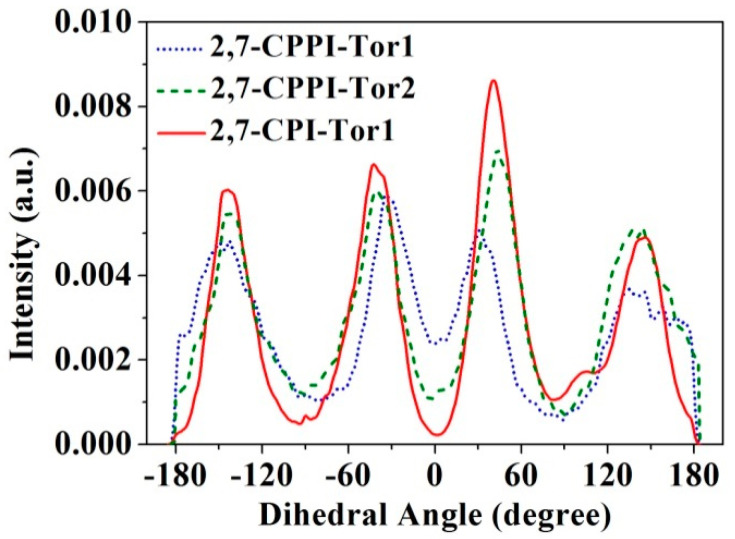
Dihedral angle profiles of the 2,7-CPPI and 2,7-CPI. (Schematic diagrams of the analyzed atomic segments are shown in Figure S3).

In addition to free volume, the local mobility of polymer chains also has a vital impact on the gas transport performances of polymers. The backbone mean square displacements (MSD) of Kapton, 2,7-CPPI and 2,7-CPI systems were calculated and shown in Figure 10. It is evident that the MSD of the three PIs decreased in the order: Kapton ˃ 2,7-CPPI ˃ 2,7-CPI. The introduction of ether bonds into the main chains of Kapton facilitated its chains mobility, while the rigid planar carbazoles in 2,7-CPPI and 2,7-CPI hindered the chains mobility. The 2,7-CPI showed lower chains mobility than 2,7-CPPI. The dihedral angle profiles of the polymer chain segments were employed to analyze the mobility of linking groups in 2,7-CPPI and 2,7-CPI, as shown in Figure 11. Compared to 2,7-CPPI, the dihedral angle peaks of the 2,7-CPI were relatively higher and narrower, demonstrating that the rotational freedoms of imide rings and carbazoles along the axis of C‒N bond were restricted [47]. This was a reason for the lower chains mobility of 2,7-CPI. In addition, the more number of hydrogen bonds formed in 2,7-CPI also contributed to its lower chains mobility. The lower chains mobility was another reason for the high barrier properties observed in 2,7-CPI. The mobility of polymer chains has a crucial effect on the channel formation for the gas molecules to permeate [48]. The poor chain mobility of 2,7-CPI reduced the degree of channel formation for gas diffusion, thus led to the high gas barrier. The high chain mobility of Kapton contributed to its high gas permeability.

### 3.8. Gas Transport Behaviour

In order to better understand the barrier performances of the three PIs, gas transport behavior including gas diffusion and solubility are investigated.

#### 3.8.1. Gas Diffusion

**Figure 12:**
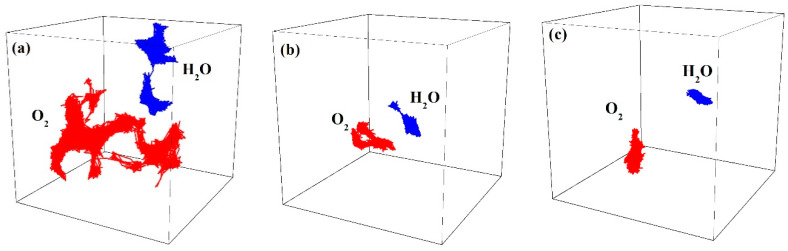
The representative trajectories of O_2_ and H_2_O in (**a**) Kapton, (**b**) 2,7-CPPI and (**c**) 2,7-CPI.

The diffusion behaviors of O_2_ and H_2_O through the PI matrices were analyzed. The representative trajectories of O_2_ and H_2_O molecules in the three PI systems during 10 ns simulation run are shown in Figure 12. Additionally, the displacements |r_i_ (t) − r_i_ (0)| of O_2_ and H_2_O are presented in Figure S11. The curves for 2,7-CPPI and 2,7-CPI have been shifted vertically for better visualization in Figure S11. As shown in Figure 12 and Figure S11, the diffusions of O_2_ and H_2_O molecules in the polymer matrix involved random oscillations within the available free volume and occasional jumps from one voids to another [49]. The length of the trajectories for O_2_ and H_2_O molecules in the three PI matrices decreased in the order: Kapton ˃ 2,7-CPPI ˃ 2,7-CPI. The jumps frequency and lengths of O_2_ and H_2_O in the three PI matrices followed the same trend (Figure S11). It also can be seen that H_2_O showed lower mobility than O_2_ in a particular PI matrix.

**Figure 13:**
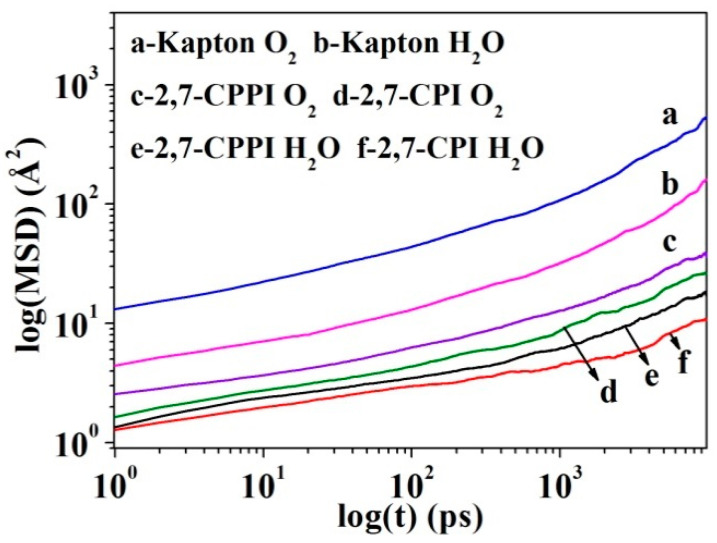
log(MSD) of O_2_ and H_2_O in Kapton, 2,7-CPPI and 2,7-CPI as a function of time.

The log(MSD) of O_2_ and H_2_O in the three PI systems was plotted as a function of time in Figure 13. The diffusion coefficient was then calculated by the Einstein’s equation in the normal diffusion regime, where the slope of the log(MSD) vs. log(*t*) curve was unity [50]. The obtained diffusion coefficients are listed in Table 4. The diffusion coefficients of O_2_ and H_2_O in the three PI matrices decreased in the order: Kapton ˃ 2,7-CPPI ˃ 2,7-CPI, which was consistent with the free volumes and chain mobility results. The 2,7-CPI had small *FFV*, poor cavities connectivity, and low chains mobility, which restricted the diffusion of H_2_O and O_2_ and resulted in the lowest diffusion coefficients. The high *FFV*, good cavities connectivity, and high chains mobility of Kapton facilitated the gas diffusion. As for a particular PI matrix, H_2_O presented lower diffusion coefficient compared with O_2_. Although the kinetic diameter of H_2_O was smaller, its diffusivity was lower than that of O_2_. This could be due to the strong interaction between polar H_2_O and PI matrix [51], which hindered the diffusion of H_2_O.

#### 3.8.2. Gas Solubility 

**Figure 14:**
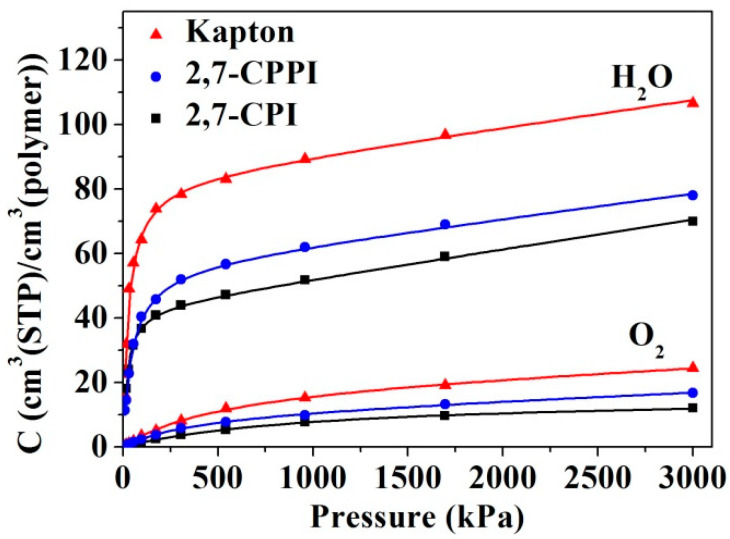
The adsorption isotherms of O_2_ and H_2_O in Kapton, 2,7-CPPI and 2,7-CPI.

To understand the gas sorption behavior, the sorption isotherms of O_2_ and H_2_O in the three PI matrices were investigated by GCMC simulations. The results are illustrated in Figure 14. The initially rapid uptake at low pressures followed by a slower gain in loading at high pressures was observed. At low pressures, the Langmuir-mode adsorption at the micro-voids was dominant, while the Henry-mode adsorption at the inter-polymer chain free volume was primary at high pressures [52]. The sorption data of O_2_ and H_2_O fitted well to dual mode sorption model and the obtained solubility coefficients are listed in Table 4. The solubility coefficients of O_2_ and H_2_O in the three PI matrices showed the trend of Kapton ˃ 2,7-CPPI ˃ 2,7-CPI. The smaller size and fewer number of cavities in 2,7-CPI resulted in fewer gas sorption sites and thereby the lowest solubility coefficients. The large size and more number of cavities in Kapton led to its highest solubility coefficients. Additionally, H_2_O presented larger solubility coefficient than O_2_ in a particular PI matrix, which was contrary to the trend of their diffusion coefficients. This was mainly due to the strong affinities between H_2_O and PI matrix [38], high critical temperature (H_2_O: 647 K, O_2_: 155 K) and small size of H_2_O, which favored the adsorption processes of H_2_O in the polymer matrix.

#### 3.8.3. Gas Permeability

According to solution-diffusion mechanism, permeability coefficient (*P*) can be obtained by the product of the diffusion coefficient and solubility coefficient. Table 4 shows the permeability coefficients of O_2_ and H_2_O in the three PIs. The simulated permeabilities of O_2_ and H_2_O in the three PIs showed similar trends as the experimental results (Table 2), validating the feasibility of simulations for gas transport analysis. The permeability coefficients of O_2_ and H_2_O in the three PIs decreased in the following the order: Kapton ˃ 2,7-CPPI ˃ 2,7-CPI, which was in agreement with the trends of diffusion and solubility coefficients. It can be seen that the reductions of diffusion and solubility coefficients of O_2_ and H_2_O in 2,7-CPI gave rise to the decreases of permeability coefficients. Consequently, the barrier performances of 2,7-CPI were enhanced. The high gas barrier of 2,7-CPI was mainly ascribed to its high crystallinity, low free volume, and poor chains mobility.

## 4. Conclusions

A intrinsic high-barrier polyimide (2,7-CPI) based on PMDA and a novel diamine (2,7-CDA) containing carbazole moiety was prepared. The 2,7-CPI exhibited high gas barrier, exceptional thermal stability, and good mechanical properties. Using molecular simulations, WAXD and PALS, the barrier performances of 2,7-CPI are compared with those of a structural analogue (2,7-CPPI) and a typical polyimide (Kapton) to reveal the barrier improvement of 2,7-CPI in relation to microstructure. The results showed that 2,7-CPI exhibited compact chain packing caused by the better coplanar structure and more number of intermolecular hydrogen bonds, which resulted in high crystallinity and low *FFV*. The Kapton showed loose chain packing resulted from the nonplanar structure and low interchain cohesion, which led to high *FFV*. Molecular simulations revealed that the chain mobility of the three PIs decreased in the order: Kapton ˃ 2,7-CPPI ˃ 2,7-CPI. The high crystallinity, low free volume, and decreased chain mobility reduced the diffusion and solubility of the gases in the polymer matrix, thus leading to the improvement of barrier performances for 2,7-CPI. The high *FFV* and chain mobility of Kapton increased the gases diffusion and solubility, thus leading to high gas permeability. The excellent comprehensive performances of 2,7-CPI suggest its promising application in micro-electronics encapsulation and high grade packaging industry.

## Figures and Tables

**Table 1:** Thermal and mechanical properties of the 2,7-CPI film.

PI	*T_g_*^a^ (°C)	*T_d5%_* (°C)	*T_d10%_* (°C)	CTE ^b^ (ppm∙K^−1^)	Tensile Strength (MPa)	Tensile Modulus (GPa)
Kapton ^c^	374	550	571	38.0	121 ± 3.0	2.3 ± 0.2
2,7-CPPI ^d^	437	556	580	2.89	143.8 ± 3.5	4.5 ± 0.2
2,7-CPI	467	550	590	3.40	134 ± 3.0	5.7 ± 0.2

^a^ Measured by DMA; ^b^ CTE within the range of 100~300 °C; ^c^ The values are obtained from Ref. [35]; ^d^ The values are obtained from Ref. [21].

**Table 2:** Barrier properties of Kapton, 2,7-CPPI and 2,7-CPI films.

PIs	WVP (g·mil·m^−2^·day^−1^)	OP (cm^3^·mil·m^−2^·day^−1^)	WVTR (g·m^−2^·day^−1^)	OTR (cm^3^·m^−2^·day^−1^)
Kapton ^a^	114.5 ± 2.12	330.7 ± 2.50	38.8 ± 0.72	112.0 ± 0.80
2,7-CPPI ^a^	0.3 ± 0.02	0.5 ± 0.03	0.1 ± 0.01	0.2 ± 0.01
2,7-CPI	0.15 ± 0.01	0.41 ± 0.02	0.05 ± 0.003	0.14 ± 0.007

^a^ The barrier properties values are obtained from Ref. [21].

**Table 3:** Physical properties and *FFV* of Kapton, 2,7-CPPI and 2,7-CPI.

PIs	Density (g.cm^−3^)	2θ (°)	*d*-Spacing (Å)	CED (J/cm^3^)	N ^a^ (H-bonds)	*R*^b^ (Å)	*FFV*^b^ (%)	*FFV*^c^ (O_2_,%)	*FFV*^c^ (H_2_O,%)	*FFV*^0^^c^ (%)
Kapton	1.42	18.64	4.76	454	0	2.60	11.50	10.48	15.80	38.51
2,7-CPPI	1.57	21.91	4.05	570	49	2.14	6.82	6.29	11.51	35.33
2,7-CPI	1.58	22.11	4.01	613	60	2.14	6.09	5.48	10.33	34.16

^a^ The number of hydrogen bonds in the simulation cells. ^b^ The free-volume parameters tested by PALS. ^c^ Free volume parameters calculated by MD simulations; *FFV*(O_2_), *FFV*(H_2_O) and *FFV*^0^ based on probe radii of 1.73 Å, 1.325 Å and 0 Å, respectively.

**Table 4:** Simulated diffusion coefficients, solubility coefficients, and permeability for O_2_ and H_2_O in Kapton, 2,7-CPPI and 2,7-CPI.

PIs	D ^a^		S ^b^		P ^c^	
	H_2_O	O_2_	H_2_O	O_2_	H_2_O	O_2_
Kapton	12.8	89.4	3.92	0.056	50.18	5.00
2,7-CPPI	3.0	6.13	0.64	0.013	1.92	0.08
2,7-CPI	2.1	5.32	0.27	0.008	0.57	0.04

^a^ Units of (10^−8^ cm^2^/s). ^b^ Units of (cm^3^(STP)·cm^−3^·cmHg^−1^). ^c^ Units of (10^−^^8^ cm^2^·cm^3^(STP) s^−1^·cm^−3^·cmHg^−1^).

## Supplementary Materials

The following are available online at https://www.mdpi.com/2073-4360/12/9/2048/s1. Figures S1–S2: Plots of density and energy versus simulation time in the NPT simulation for PIs. Figure S3: Schematic diagrams of the analyzed atomic segments for the dihedral angle analysis. Figures S4–S6: ^1^H NMR, ^13^C NMR spectra and mass spectrum of DPNA, DPNN_3_, 2,7-CDN. Figure S7: Mass spectrum of 2,7-CDA. Figure S8: FT-IR spectra of DPNA, DPNN_3_, 2,7-CDN, 2,7-CDA, and 2,7-CPI. Figures S9–S10: TGA curve and photo image of 2,7-CPI film. Figure S11 and Table S1: The positron lifetime data and displacement of O_2_ and H_2_O for Kapton, 2,7-CPPI, and 2,7-CPI.
