# Supplementary material for: High-Barrier Polyimide Containing Carbazole Moiety: Synthesis, Gas Barrier Properties, and Molecular Simulations
Source: Polymers (Basel). 2020 Sep 8;12(9):2048. doi: 10.3390/polym12092048 (PMC7565694; doi:10.3390/polym12092048)
Supplement: Supplementary file 1 [file polymers-12-02048-s001.pdf]

Supporting Information

High-barrier polyimide containing carbazole moiety: synthesis, gas barrier properties and molecular simulations

Yiwu Liu[a], Ao Tang[a], Jinghua Tan[a], Xianqing Zhao[a], Chengliang Chen[a], Ding Wu[a], Yuhui Li[a], Pan He[a], Hailiang Zhang[b]

a. National and Local Joint Engineering Center of Advanced Packaging Materials R & D Technology, Key Laboratory of Advanced Packaging Materials and Technology of Hunan Province, School of Packaging and Materials Engineering, Hunan University of Technology, Zhuzhou 412007, P. R. China

b. Key Laboratory of Polymeric Materials and Application Technology of Hunan Province, Key Laboratory of Advanced Functional Polymer Materials of Colleges, Universities of Hunan Province, College of Chemistry, Xiangtan University, Xiangtan 411105, P. R. China

*To whom all correspondence should be addressed. E-mail:

tjh@hut.edu.cn

This PDF file includes:

Table S1

Figures S1-S11

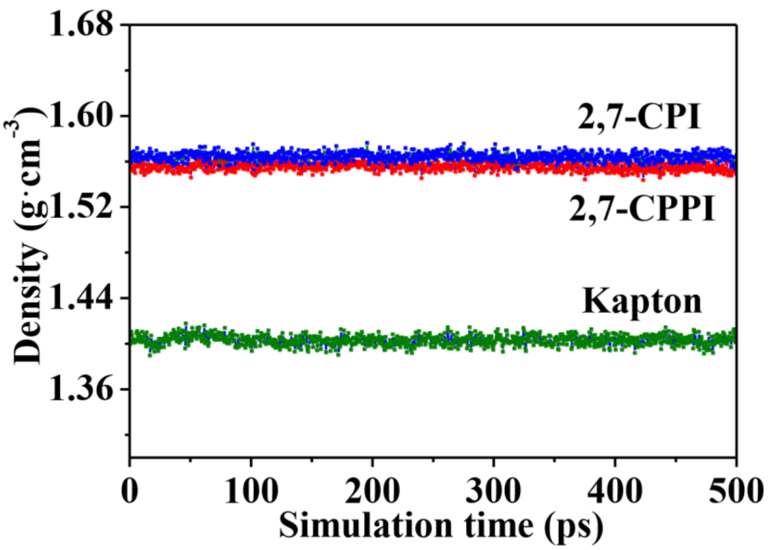

Figure S1 Plots of density versus simulation time in the NPT simulation for Kapton, 2,7-CPPI and 2,7-CPI.

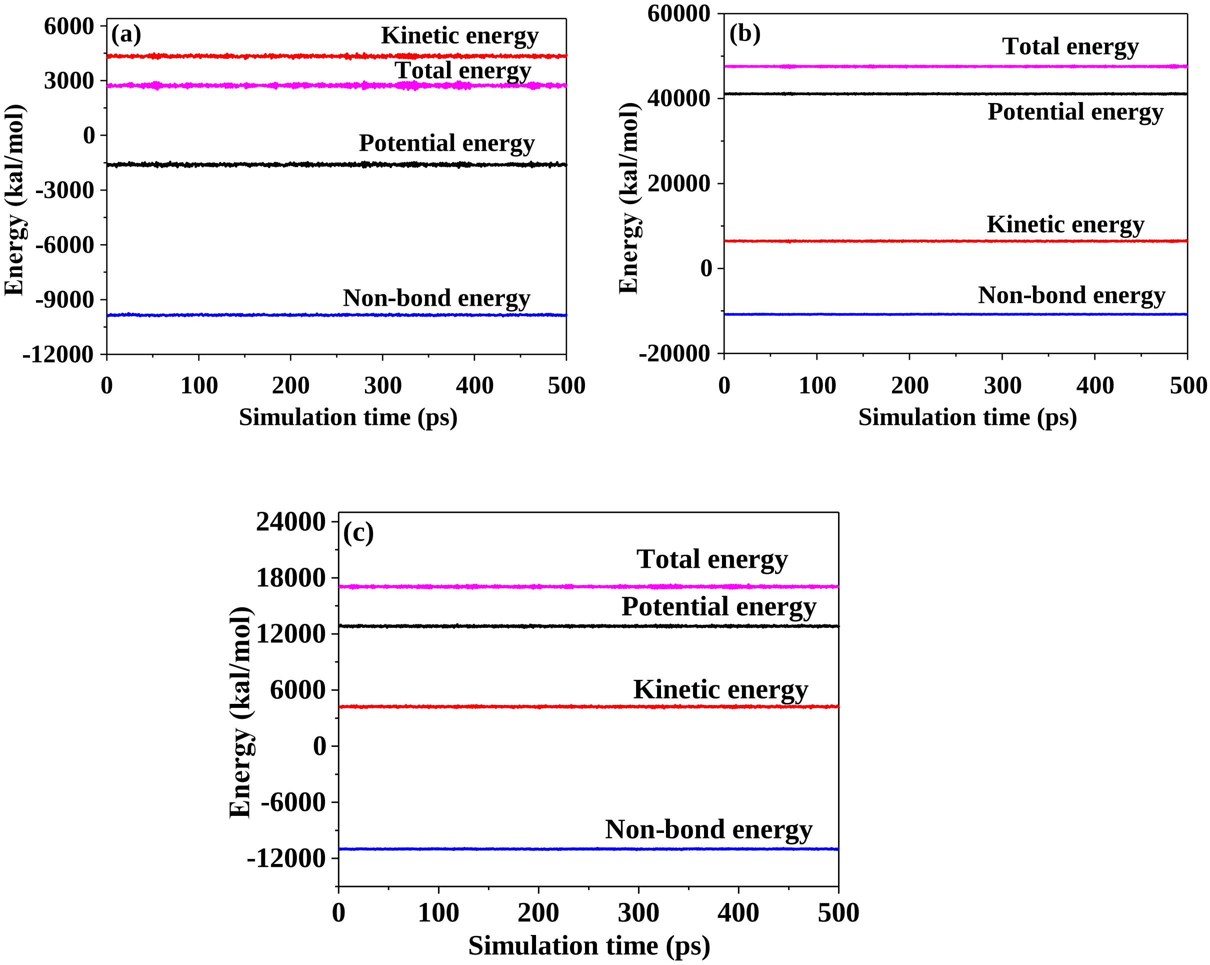

Figure S2 Plots of energy versus simulation time in the NPT simulation for (a) Kapton, (b) 2,7-CPPI and (c) 2,7-CPI.

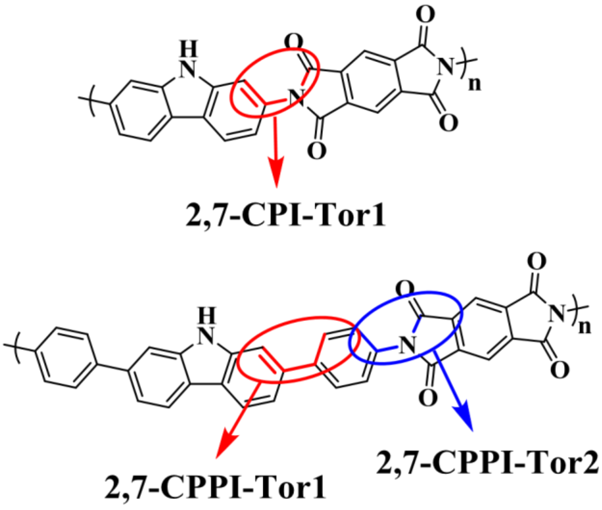

Figure S3 Schematic diagrams of the analyzed atomic segments for the dihedral angle analysis.

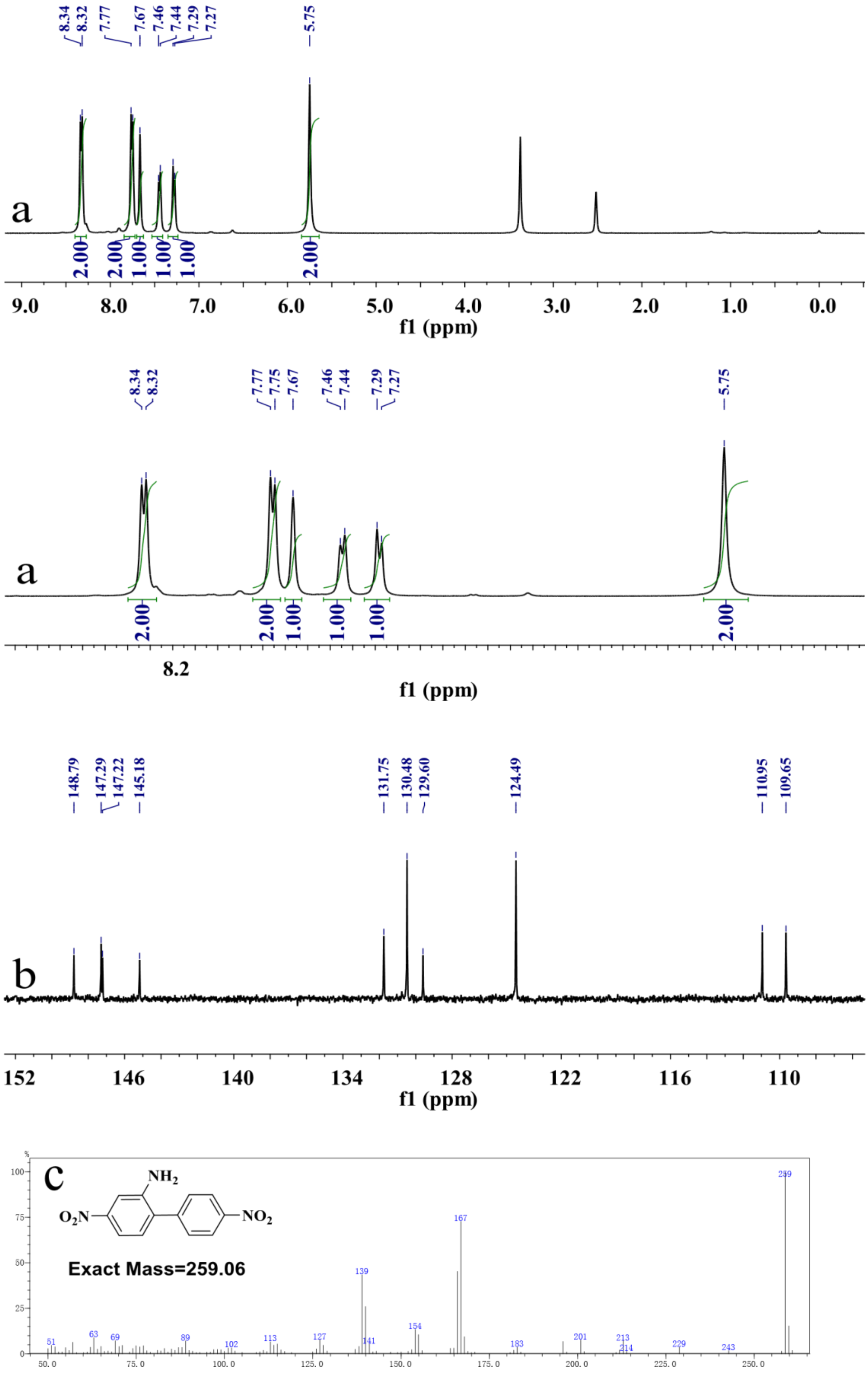

Figure S4 ^{1}H NMR (a), ^{13}C NMR (b) and MS (c) spectra of DPNA.

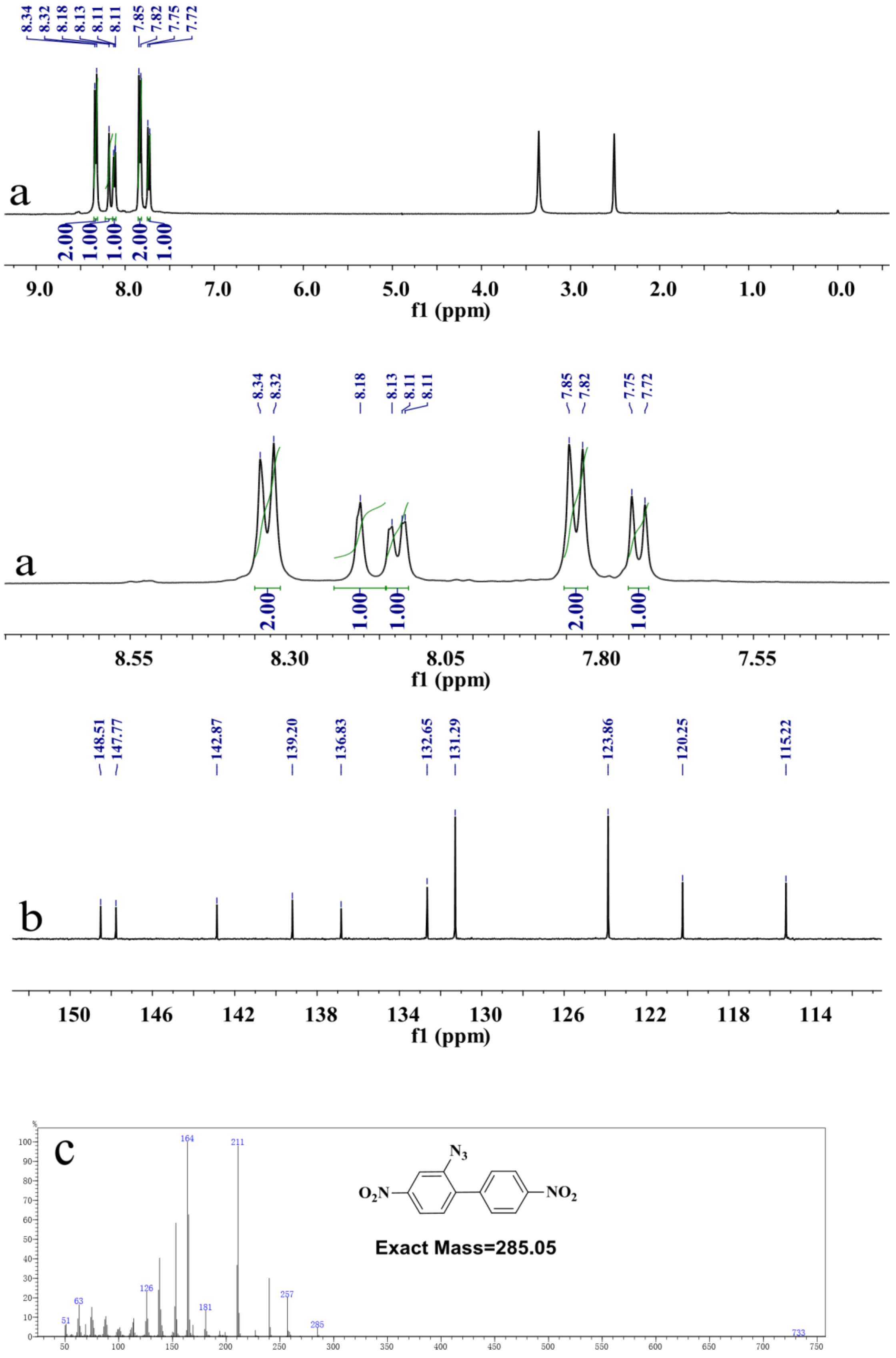

Figure S5 ^{1}H NMR (a), ^{13}C NMR (b) and MS (c) spectra of $DPNN_3$.

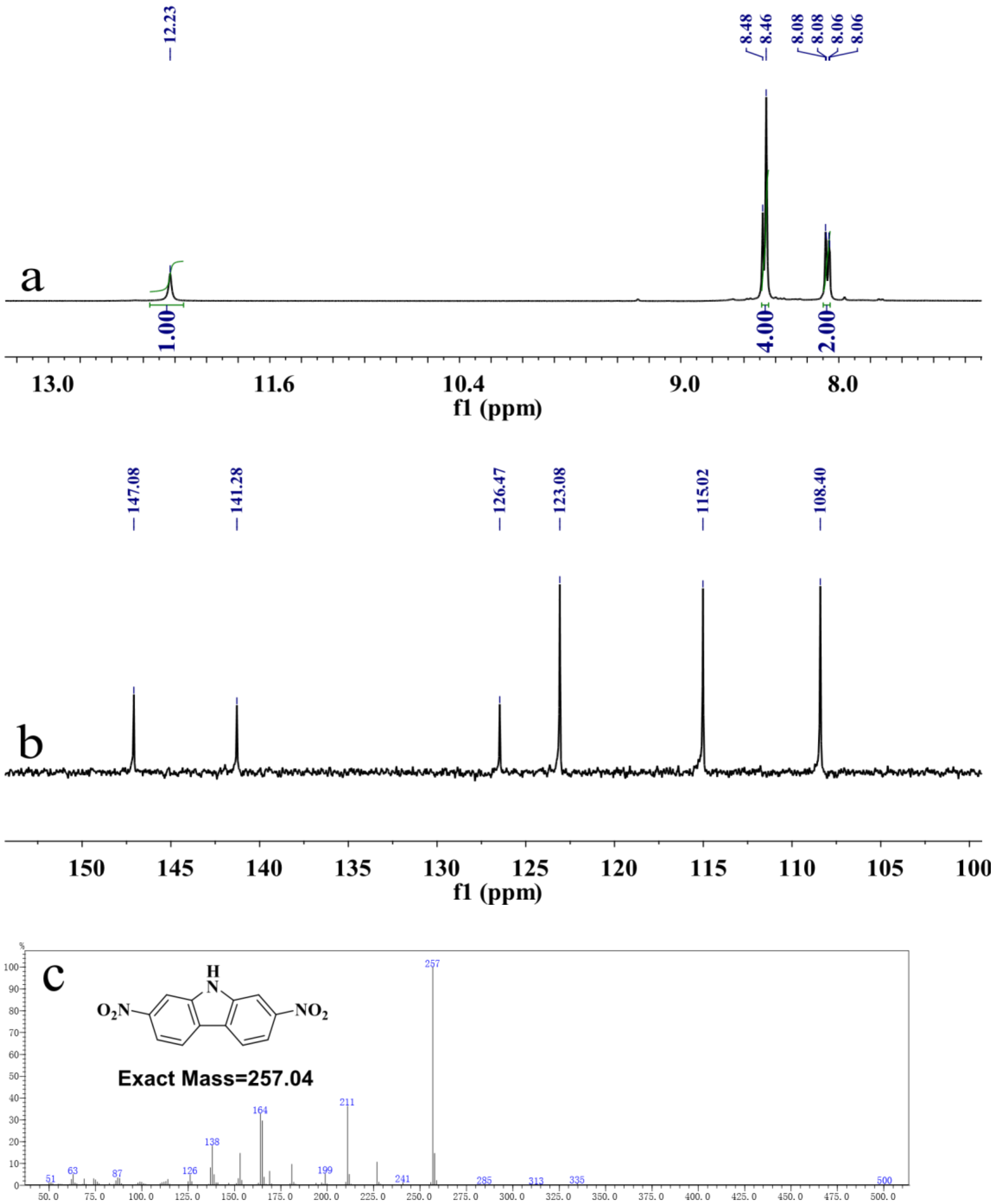

Figure S6 ^{1}H NMR (a), ^{13}C NMR (b) and MS (c) spectra of 2,7-CDN.

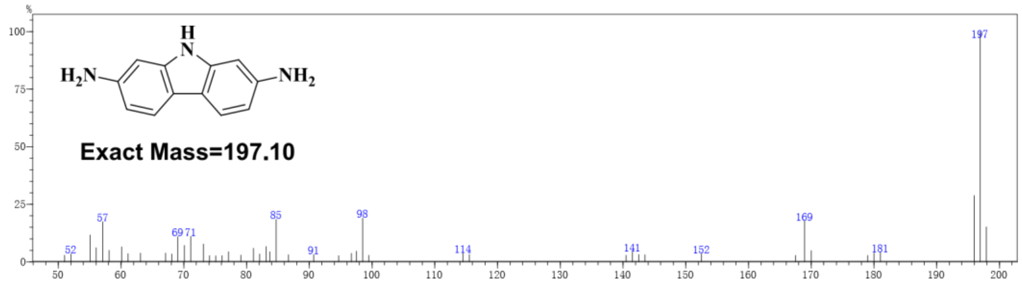

Figure S7 The MS spectrum of 2,7-CDA.

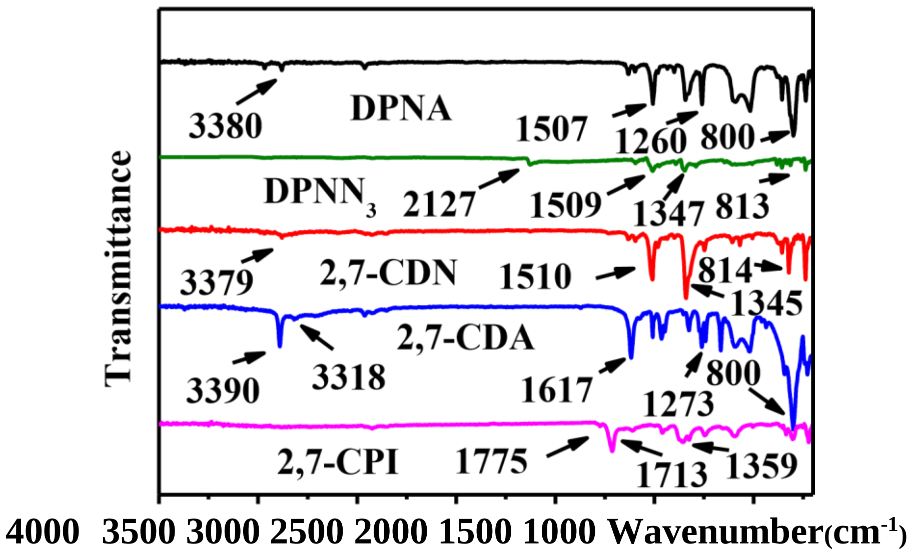

Figure S8 FT-IR spectra of DPNA, $DPNN_3$, 2,7-CDN, 2,7-CDA and 2,7-CPI.

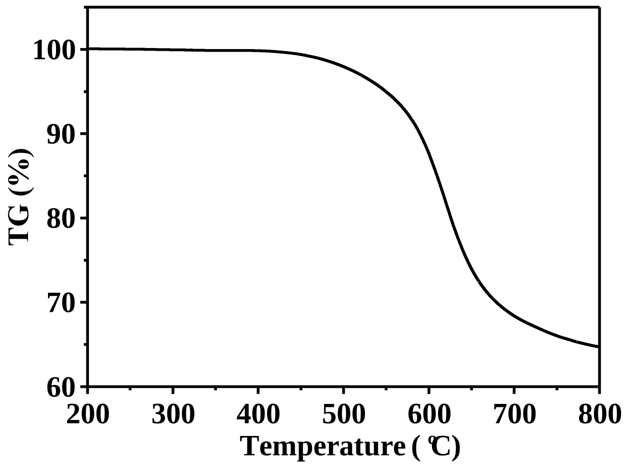

Figure S9 TGA curve of the 2,7-CPI film.

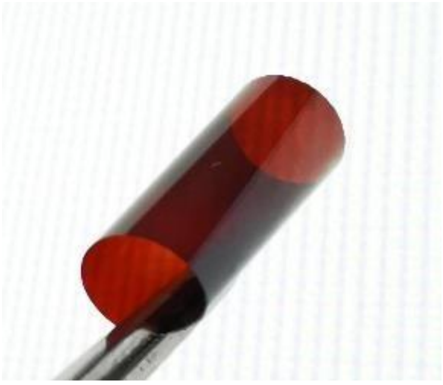

Figure S10 Photo image of the flexible 2,7-CPI film.

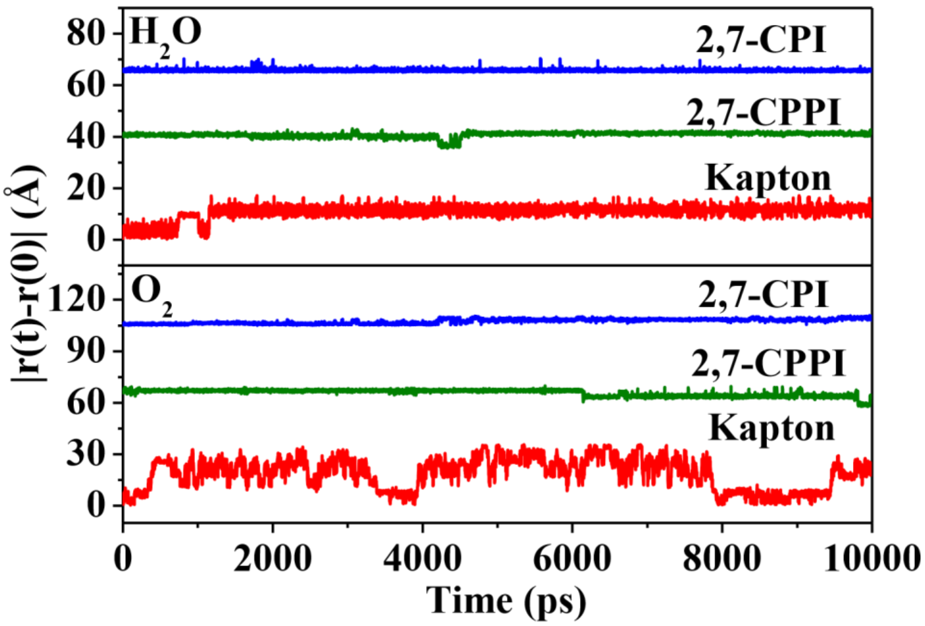

Figure S11 Displacement of O_2 and H_2O from their initial positions in Kapton, 2,7-CPPI and 2,7-CPI.

Table S1 The positron lifetime data of Kapton, 2,7-CPPI and 2,7-CPI films.

**PIs**	τ_1**(ns)**	I_1**(%)**	τ_2**(ns)**	I_2**(%)**
Kapton[a]	0.17	13.0	0.38	86.8
2,7-CPPI[a]	0.17	7.4	0.34	92.4
2,7-CPI	0.13	17.3	0.34	82.5

[a] The positron lifetime data are obtained from Ref. [1].

References

1. Liu, Y.; Huang, J.; Tan, J.; Zeng, Y.; Liu, J.; Zhang, H.; Pei, Y.; Xiang, X.; Liu, Y. Intrinsic high-barrier polyimide with low free volume derived from a novel diamine monomer containing rigid planar moiety. *Polymer* **2017**, *114*, 289-297.
